# Seed Dormancy, Seedling Establishment and Dynamics of the Soil Seed Bank of *Stipa bungeana* (Poaceae) on the Loess Plateau of Northwestern China

**DOI:** 10.1371/journal.pone.0112579

**Published:** 2014-11-14

**Authors:** Xiao Wen Hu, Yan Pei Wu, Xing Yu Ding, Rui Zhang, Yan Rong Wang, Jerry M. Baskin, Carol C. Baskin

**Affiliations:** 1 State Key Laboratory of Grassland Agro-ecosystems, College of Pastoral Agriculture Science and Technology, Lanzhou University, Lanzhou, 730020, China; 2 Department of Biology, University of Kentucky, Lexington, Kentucky 40506-0225, United States of America; 3 Department of Plant and Soil Sciences, University of Kentucky, Lexington, Kentucky 40546-0312, United States of America; Shandong University, China

## Abstract

Studying seed dormancy and its consequent effect can provide important information for vegetation restoration and management. The present study investigated seed dormancy, seedling emergence and seed survival in the soil seed bank of *Stipa bungeana*, a grass species used in restoration of degraded land on the Loess Plateau in northwest China. Dormancy of fresh seeds was determined by incubation of seeds over a range of temperatures in both light and dark. Seed germination was evaluated after mechanical removal of palea and lemma (hulls), chemical scarification and dry storage. Fresh and one-year-stored seeds were sown in the field, and seedling emergence was monitored weekly for 8 weeks. Furthermore, seeds were buried at different soil depths, and then retrieved every 1 or 2 months to determine seed dormancy and seed viability in the laboratory. Fresh seeds (caryopses enclosed by palea and lemma) had non-deep physiological dormancy. Removal of palea and lemma, chemical scarification, dry storage (afterripening), gibberellin (GA_3_) and potassium nitrate (KNO_3_) significantly improved germination. Dormancy was completely released by removal of the hulls, but seeds on which hulls were put back to their original position germinated to only 46%. Pretreatment of seeds with a 30% NaOH solution for 60 min increased germination from 25% to 82%. Speed of seedling emergence from fresh seeds was significantly lower than that of seeds stored for 1 year. However, final percentage of seedling emergence did not differ significantly for seeds sown at depths of 0 and 1 cm. Most fresh seeds of *S. bungeana* buried in the field in early July either had germinated or lost viability by September. All seeds buried at a depth of 5 cm had lost viability after 5 months, whereas 12% and 4% seeds of those sown on the soil surface were viable after 5 and 12 months, respectively.

## Introduction

Soil erosion is the main cause of land degradation in arid and semiarid regions, and it is a widespread problem on the Chinese Loess Plateau [1]. One way to restore degraded soils and reduce soil erosion is by revegetation [2], [3]. The first step in any program of rehabilitation of soils degraded by erosion is to select the most suitable species to use for revegetation, based on the capacity of the seeds to germinate and the seedlings to become established [4], [5].

*Stipa bungeana* Trin. (Poaceae) is a perennial grass that mainly occurs in semi-arid areas of the temperate steppe zone in Eurasia. The species is widely distributed on the Loess Plateau and other areas of western China. It is the main wild forage species in natural grasslands of northwestern China and also plays important roles in protecting the soil from erosion and reducing water loss by runoff. Due to its environmental benefits and economic value, Cheng *et al.* [6] and Hu *et al*. [7] suggested that *S. bungeana* is a potential key species for revegetation of degraded land on the Loess Plateau.

Seed dormancy is the failure of viable seeds to germinate in a specified period of time under conditions suitable for their germination after they become nondormant [8], [9]. Dormancy could prevent or delay germination even under favorable conditions, thus enabling seeds to accumulate in the soil seed bank and preventing plants from expending their entire reproductive outputs at a given time [10]. As such, then, seed dormancy is expected to be important in optimizing the timing of germination to maximize seedling establishment. Although *S. bungeana* produces up to 1430 seeds m^−2^ in typical *S. bungeana*-dominated rangeland, only a low portion of them germinate in the field; thus, few seedlings became established following seed dispersal [7]. Two reasons may contribute to low seedling establishment: 1) fresh seeds of *S. bungeana* exhibit primary dormancy, which prevents seed germination immediately after dispersal; and 2) environmental conditions during the dispersal season prevent seeds from germinating. In the first case, no seeds, or only a small portion of them, germinate even under otherwise favorable conditions until dormancy release. Thus, we expected that primary dormancy plays a role in regulating the time of seed germination and seedling recruitment in the field. Hu *et al*. [7] showed that germination of *S. bungeana* seeds was inhibited by light and sensitive to water stress, implying that most seeds would germinate slowly or not at all on the soil surface. Thus, seed burial in the soil may play a key role in determining whether they can germinate after dispersal.

The effects of temperature [11], light [11], water stress [7] and burial depth [7] on germination and of fungicide pretreatment on seed survival in the field have been determined for seeds of *S. bungeana* stored (afterripened) in the lab for 1 year. However, no studies have been done to test for seed dormancy in *S. bungeana* and its underlying mechanism, and consequently its effect on seedling emergence and seed survival in the soil. Moreover, seeds stored in the lab for 1 year were used in previous studies. As reported by Baskin and Baskin [12], results from studies initiated after seeds have been stored dry may have little ecological relevance. That is, the germination responses of seeds may have changed through time, and thus interpretation of results obtained using stored seeds may differ from fresh seeds dispersed in the natural environment.

Thus, the aims of this study were to determine: 1) whether fresh seeds are dormant and if so why; 2) the effect of storage condition on seed dormancy of fresh seeds; 3) the effect of seed dormancy on seedling emergence in relation to sowing depth; and 4) seed dormancy and survival of fresh seeds in relation to burial depth and duration in the field.

## Materials and Methods

### Seed collection

*Stipa bungeana* flowers in the early May, and seeds mature and are dispersed in late June in the study area. The dispersal unit of *S. bungeana* is a caryopsis tightly enclosed by the palea and lemma. It is 5–6 mm in length and 0.7–1.0 mm in width. Hereafter, the dispersal unit of *S. bungeana* will be referred to as a seed.

*S. bungeana* seeds were collected on 23 June 2012 and 28 June 2013 from a field on the Yuzhong Campus of Lanzhou University, Gansu Province (35°57′N, 104°10′E). The mean annual temperatures is 6.7°C and mean annual rainfall 350 mm, most of which falls from July to September. The soils consist of silt (66.9%), clay (20.8%) and sand (12.3%), and natural vegetation is dominated by *S. bungeana*. Other species growing at this site include *Achnatherum inebrians*, *Artemisia* spp., *Glycyrrhiza* spp. and *Lespedeza davurica*. Infructescences with ripe seeds were collected from several hundred plants and taken to the laboratory, where the seeds were separated from them, cleaned and dried at room temperature for one week (RH, 20–35%, 18–25°C) and stored at 4°C until used in experiments. The experiments were conducted within two weeks after seed collection except for the one on seedling emergence.

### Seed viability

Viability of fresh seeds collected in 2012 and 2013 was determined. Seeds were soaked in distilled water for 12 h, after which hulls and half of endosperm were removed. Then the remaining part of the seed containing the embryo was soaked in 1% tetrazolium phosphate-buffer solution for 6–8 hours at 30°C in the dark. Seeds with embryos that stained red were considered to be viable and those with unstained embryos nonviable. For fresh seeds collected in 2012 and 2013, four replicates of 50 seeds were tested.

### Effect of light and temperature on germination

The aim of this experiment was to determine whether fresh seeds are dormant. Fresh seeds were tested at four constant (10°C, 15°C, 20°C and 25°C) and three alternating (10/20°C, 15/25°C and 20/30°C) temperature regimes (12 h/12 h). At each temperature, seeds were incubated at a 12 h/12 h daily photoperiod (hereafter light) or in continuous darkness. For treatments in light, seeds were exposed to light produced by white fluorescent tubes with a photon irradiance of 60 µmol·m^−2^·s^−1^ (400–700 nm). For continuous darkness, Petri dishes were covered with two layers of aluminum foil, and seeds were monitored for germination (root emergence) daily under a LED green safe light (520 nm±10 nm, Sanpai, Shanghai, China). Photon irradiance at Petri dishes level was 10 µmol·m^−2^·s^−1^, as determined by use of a quantum sensor (LI-190SA) connected to a LI-6400 portable photosynthesis system (LI-COR, USA). For each treatment, four replicates of 50 seeds each were placed in 11-cm-diameter Petri dishes on two sheets of filter paper (Shuangquan, Hangzhou) moistened with 8 mL of distilled water. Seeds incubated in both light and dark were examined for germination daily for 14 days, and any seedlings present were removed from the Petri dishes.

### Effect of hulls, half-endosperm removal and scarification with NaOH on germination

The aim of this experiment was to determine the role of hulls and endosperm in controlling seed dormancy and effect of NaOH on breaking dormancy. The mechanical removal experiment consisted of a control (caryopsis with intact lemma and palea) and three treatments: 1) hulls (lemma and palea) removed; 2) hulls removed and put back in their original position enclosing the caryopsis; and 3) half of endosperm (and enclosing portions of hulls) removed from the endosperm-end of the seed without damaging the embryo. Before performing the removal treatments, seeds were immersed in distilled water for 12 h, and then the hulls were removed from the caryopses using forceps. For treatment 2, the hulls were removed from the seed and then re-attached loosely to their original position starting at the embryo end. For treatment 3, half of the endosperm (along with enclosing portions of hulls) was removed from the endosperm-end of the seed with a scalpel.

For scarification with NaOH, seeds (with hulls) were soaked in 50 mL of a 30% NaOH solution at 20°C for 20, 40 or 60 min. Then the seeds were rinsed thoroughly with tap water five times and allowed to dry on filter paper for 48 h on the laboratory bench. For each treatment, four replicates of 50 seeds were used for testing germination at 20°C in dark. Seeds without pretreatment were used as a control. Germination was monitored daily for 14 days as described above.

### Effect of dry storage on seed dormancy

To determine the effect of storage duration and temperature on seed dormancy, fresh seeds were placed in a paper bag and stored in darkness at 5°C and at 20°C for 1, 3 and 6 months. After each treatment, germination was tested in light and in darkness at 20°C. There were four replicates of 50 seeds per treatment. Germination was monitored daily for 14 days as described above.

### Effect of fluridone, GA_3_ and KNO_3_ on seed germination

To determine the role of plant growth regulator and potassium nitrate (KNO_3_) in controlling seed dormancy of *S. bungeana*, seeds were treated with gibberellic acid (GA_3_, Sigma, China); fluridone (FLU, Sigma, China), an inhibitor of abscisic acid (ABA) biosynthesis, or KNO_3_. Fluridone and GA_3_ each were dissolved in 2 mL ethanol prior to dilution in water, and the final concentration of FLU and GA_3_ was 200 µM. A preliminary experiment showed that a low concentration of ethanol did not affect germination of *S. bungeana* seeds. The potassium nitrate solution was 1 mM. Seeds incubated in distilled water were used as control. For each treatment, four replicates of 50 seeds were incubated at 20°C in dark. Germination was monitored daily for 14 days as described above.

### Seedling emergence in field

To determine the effect of seed dormancy on seedling emergence, the seedling emergence experiment was conducted at the Yuzhong Campus from 16 July 2013 to 12 September 2013. Seeds that had been stored dry at 20°C for 1 year and fresh seeds collected on 28 June 2013 were used in this experiment. There were two seed lots (fresh, afterripened) × three burial depths (0, 1, 5 cm) in a completely randomized design. Seeds were sown on 16 July directly in PVC pots (15 cm in diameter, 11 cm in height) that were buried in the field with the rim 5 cm above the soil surface. Soil level in the pot was even with the soil surface. To avoid seed contamination, soil was passed through a 0.5 mm-mesh wire sieve to remove any *S. bungeana* seeds present in it. Then, the soil was placed in the pots to the desired depth, and seeds were placed at this depth and covered with soil. The pots were covered with nylon mesh to prevent animal predation and contamination by extraneous seeds of *S. bungeana*. Ten replicates of 50 seeds each were used for each treatment. Seedling emergence was monitored weekly for 8 weeks, and any seedlings present were counted and removed. The vegetation surrounding the pots was removed by hand every week. The speed of seedling emergence was calculated using the emergence index (*EI*):
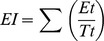
where Et is the number of seeds emerged on tth week, and Tt is the weeks of seedling emergence from sowing [13].

### Seed burial experiment

To determine the effect of burial depth and burial duration on seed survival and seed dormancy of *S. bungeana*, seeds collected on 23 June 2012 were put into 96 15 cm×10 cm nylon mesh bags and buried at the Yuzhong Campus of Lanzhou University on 5 July 2012. The permeable nylon fabrics allowed movement of water, air and microbes between inside and outside of bags. The burial site is about 300 m from the seed collection site and has a sparse vegetation cover. The vegetation within and surrounding burial site was removed before burial. Forty-eight bags with 50 seeds each were placed on the soil surface (0 cm) and 48 at a soil depth of 5 cm. They were arranged in a randomized complete block design. For 0 cm burial depth, the bags were fixed to the ground by iron nails so that each bag was in contact with the soil and won’t move to the other soil profile. Physical removal of weeds within burial site was applied every week during experimental period. Six bags each were retrieved from 0 cm and 5 cm burial depths on 28 July, 30 August, 30 September, 31 October and 30 December 2012 and on 2 March, 9 May and 5 July 2013. For each of these dates, the seeds in each of 12 bags were put into one 11-cm-diameter Petri dish with two layers of filter paper moistened with 8 mL of distilled water and incubated in darkness at 20°C. Thus, there were six replications each for the 0 cm and 5 cm burial depth treatments. The number of germinated seeds was counted after 14 days. Germination of 6 replicates of 50 seeds was tested before burial as described above. For all germination tests, seeds failed to germinate were tested for viability.

### Temporal changes in soil seed bank size

To determine soil seed bank size in the field, soil samples were taken on 28 July and 28 August, 2012 and 2 March and on 20 May, 2013 on the Yuzhong Campus of Lanzhou University. The sampling site is 2000 m^2^ and dominant plants were *S. bungeana* and *Medicago sativa*. Other species at the site included *Achnatherum inebrians* and *Artemisia* spp. Sixteen 1 m×1 m quadrats were haphazardly established at the study site for each sampling time. In each quadrat, five soil subsamples 0–5 cm deep were collected using a 10 cm×10 cm×5 cm soil sampler, mixed together, air dried and sieved through a 0.5 mm sieve. Seeds of *S. bungeana* were separated from the litter and incubated in 11-cm-diameter Petri dishes with two layers of filter paper moistened with 8 mL of distilled water in darkness at 20°C. Germination percentages were determined after 14 days of incubation, and seeds that failed to germinate were tested for viability.

### Statistical analysis

A two way ANOVA at a significance level of *P*<0.05 was used to analyze the effect of light, temperature and their interaction on seed germination and of burial time, burial depth and their interaction on seed viability and seed dormancy. Duncan’s multiple range tests was used to compare means of germination percentage between treatments when significant differences were found. Independent t-test was used to compare the mean viability of seeds collected in 2012 and 2013. Germination percentage data were arcsine transformed to increase homogeneity of variance prior to analysis, but nontransformed data are shown in all figures and in tables. All analyses were conducted in SPSS 15.0 software.

## Results

### Seed viability

Fresh seeds collected in 2012 and 2013 showed no significant difference in terms of seed viability which was 85±4.6% and 87±3.7%, respectively.

### Effect of light and temperature on germination

**Figure 1:**
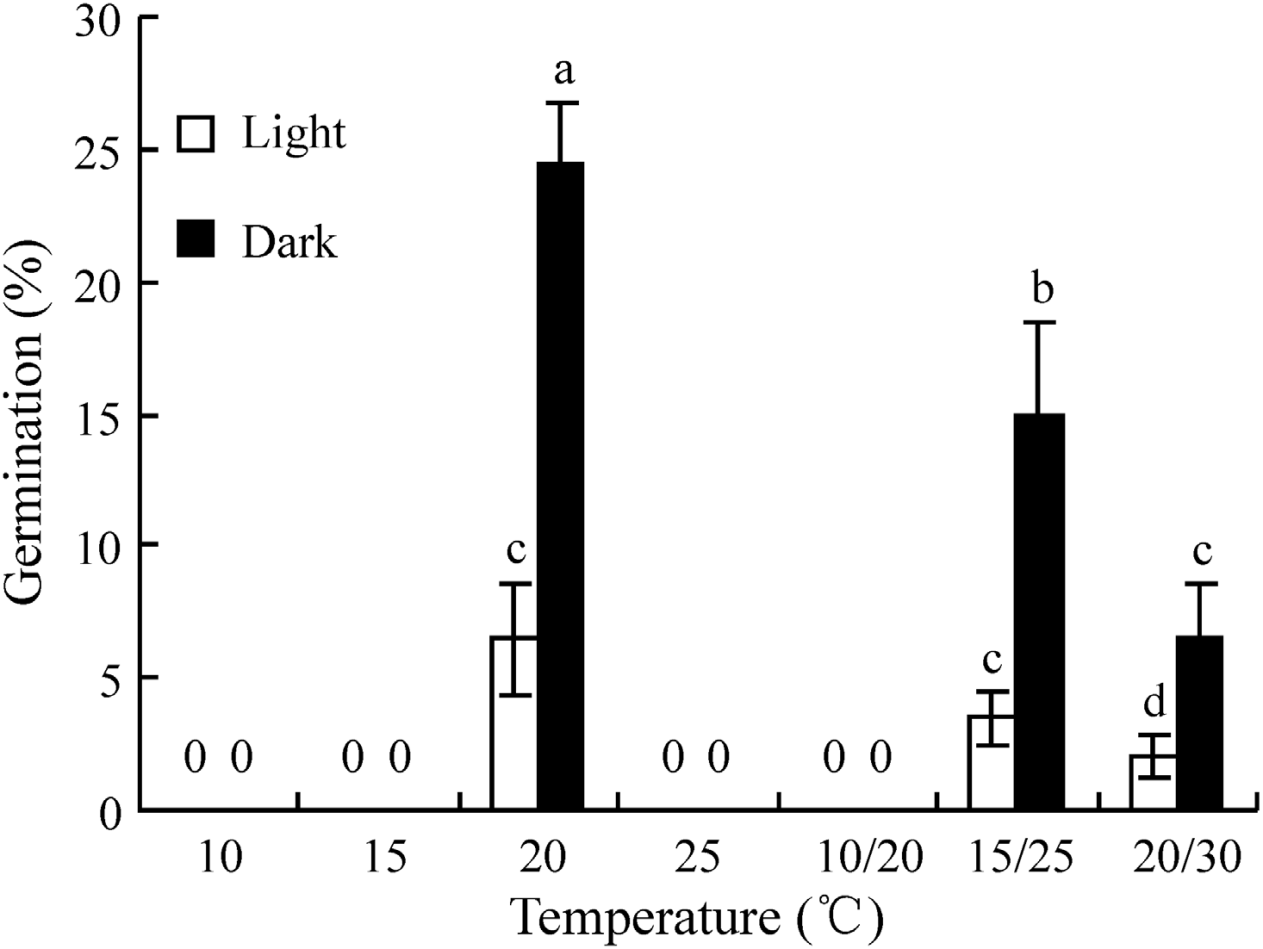
Effect of temperature on germination of fresh seeds of *Stipa bungeana* in a 12 h/12 h photoperiod and in dark. Different letters indicate significant difference (P<0.05) among all treatments (n = 4).

Light, temperature and their interaction had significant effects on germination (Fig. 1, Table 1). Light inhibited germination at the three temperatures at which germination occurred. The highest germination was 25%, in darkness at 20°C, and only 7% of the seed germinated in light. Seeds germinated to significantly lower percentage at 15/25°C and 20/30°C in both darkness and light. No seeds germinated at 10, 15, 25 or 10/20°C in darkness or in light.

**Table 1:** Two way ANOVA of the effects of temperature, light and their interaction on germination of fresh seeds of *Stipa bungeana* (n = 4).

Source	Sum of Squares	df	F	P-value
Temperature(T)	1790	6	39.2	.000
Light(L)	330	1	43.4	.000
T * L	623	6	13.6	.000

### Effect of hulls, half-endosperm removal and scarification with NaOH on seed germination

**Figure 2:**
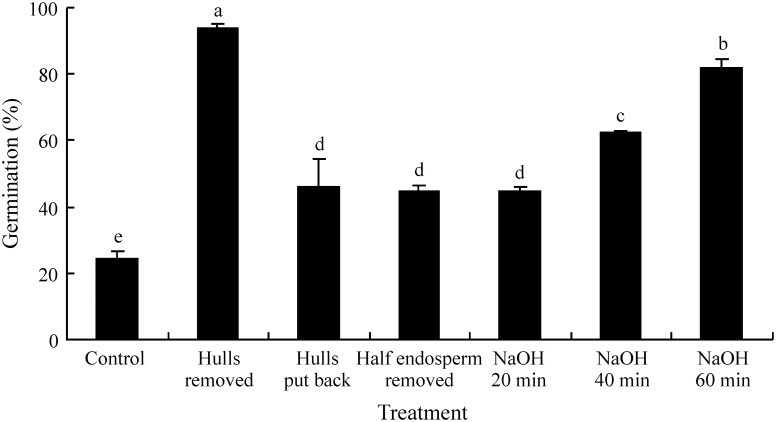
Effect of hulls, half-endosperm removal and NaOH scarification on germination of fresh seeds of *Stipa bungeana* at 20°C in dark. Different letters indicate significant difference (P<0.05) among all treatments (n = 4).

Hulls, endosperm and scarification with NaOH had a significant effect on release of seed dormancy (P<0.05, Fig. 2). Twenty-five percent of fresh seeds (with hulls) without pretreatment germinated, and dormancy was completely released when the hulls were removed. However, seeds in which the hulls were put back to their original position germinated to a significantly lower percentage (46) than those hulls removed (94). Removal of half of the endosperm increased germination from 25% to 45% (P<0.05). Seeds treated with NaOH for 20, 40 and 60 minutes germinated to 45, 63 and 82%, respectively (Fig. 2).

### Effect of dry storage on dormancy break

**Figure 3:**
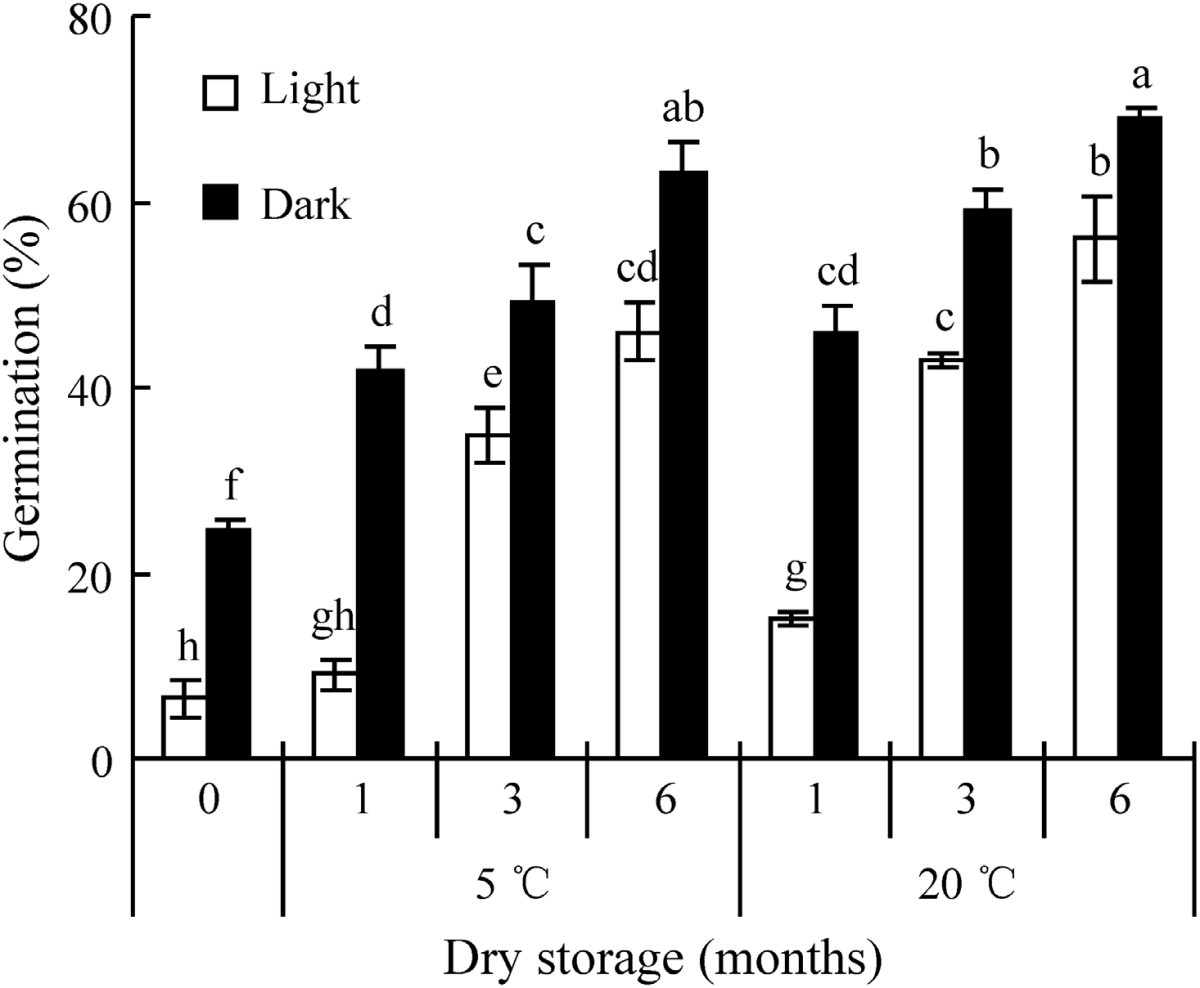
Effect of dry storage (afterripening) on germination of seeds of *Stipa bungeana* in a 12 h/12 h photoperiod and in dark at 20°C. Different letters indicate significant difference (P<0.05) among all treatments (n = 4).

Storage at 5°C and 20°C significantly increased germination percentages in light and in dark. However, there was little difference in afterripening at the two storage temperatures, although seeds afterripened of 20°C for 3 months germinated to significantly higher percentage in light and dark than those afterripened at 5°C, and seeds stored at 20°C for 6 months germinated to a significantly higher percentage in light than those afterripened at 5°C (Fig. 3).

### Effect of fluridone, GA_3_ and KNO_3_ on germination

**Figure 4:**
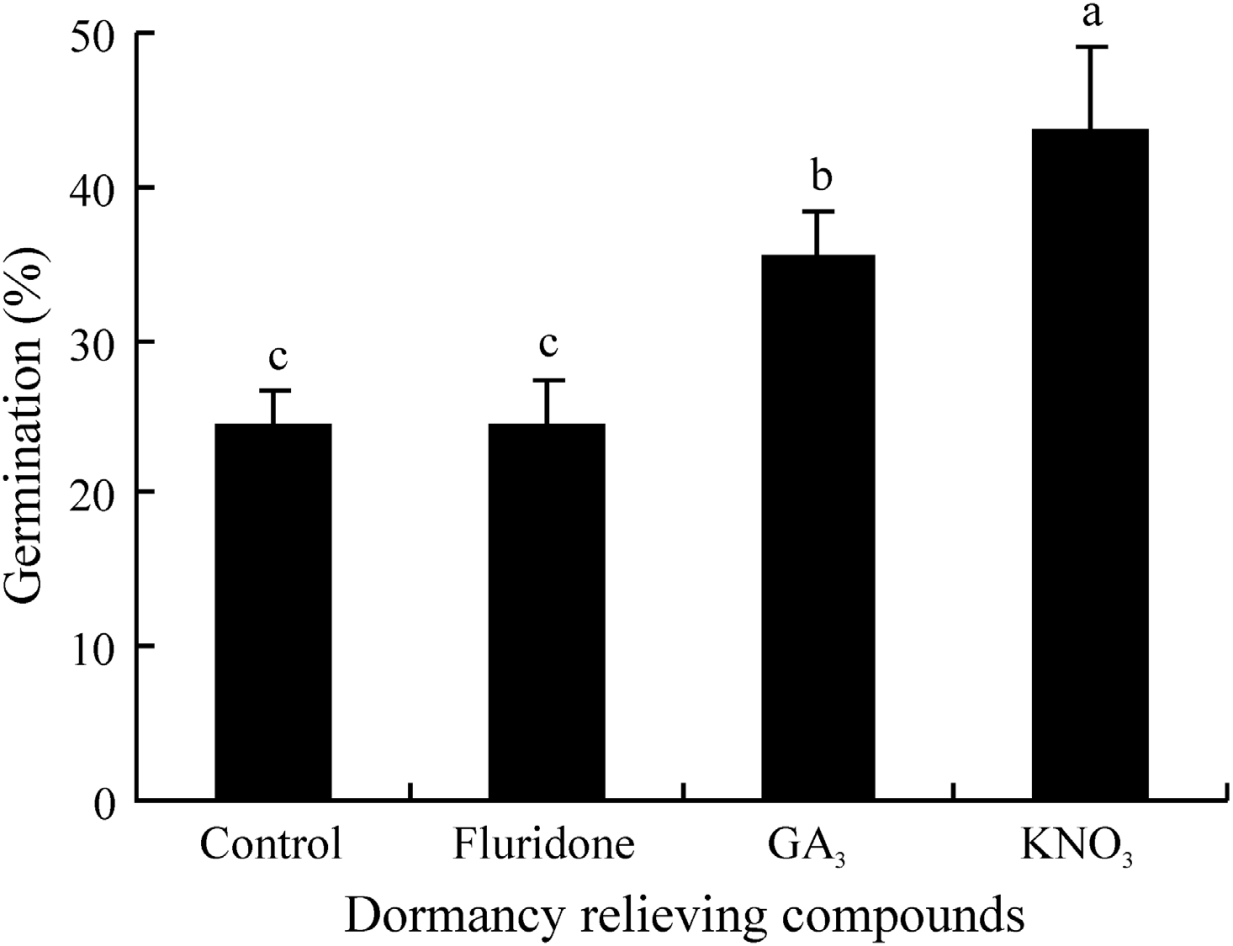
Effect of fluridone, GA_3_ and KNO_3_ on germination of fresh seeds of *Stipa bungeana* in dark at 20°C. Different letters indicate significant difference (P<0.05) among all treatments (n = 4).

GA_3_ and KNO_3_ significantly increased germination from 25% to 36% and 44%, respectively (P<0.05), but FLU had no effect. Germination percentage was significantly higher for seeds treated with KNO_3_ than for those treated with GA_3_ (Fig. 4).

### Effect of dry storage and burial depth on seedling emergence

**Figure 5:**
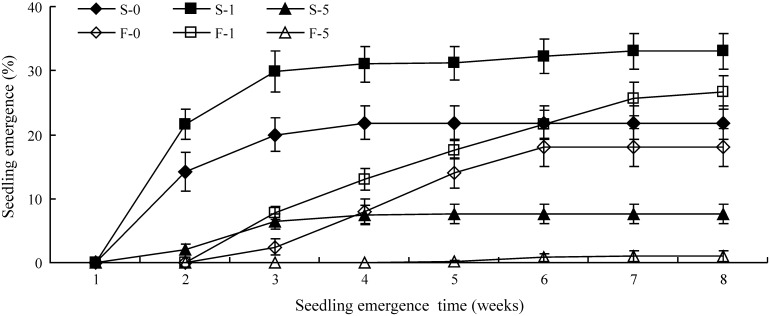
Seedling emergence from fresh (F) and one-year-stored (S) seeds of *Stipa bungeana* at 0, 1 and 5 cm sowing depths. F-0, F-1 and F-5 indicate fresh seeds sown at a depths of 0, 1 and 5 cm, respectively (n = 10). S-0, S-1 and S-5 indicate seeds stored for one year and then sown at depths of 0, 1 and 5 cm, respectively (n = 10).

Percentage and speed of seedling emergence in the field varied with burial depth and seed lot (fresh vs. one-year-stored seeds) (Fig. 5, Table 2). At 0, 1 and 5 cm burial depths, seeds stored one year had a significantly higher emergence speed (higher emergence index) than fresh seeds. Most seeds stored for 1 year germinated within 3 weeks after sowing. However, fresh seeds had just begun to germinate by the third week, and they continued to do so for another 4–5 weeks. On the other hand, there was no significant difference in final seedling emergence between stored and fresh seeds sown at 0 cm and 1 cm (Table 2). In contrast, seedling emergence percentage at 5 cm was significantly higher for stored seeds than for fresh seeds, but emergence percentages were <10% for both burial depths. The highest final seedling emergence percentage was for 1 cm burial depth, with 33% for one year stored seeds and 27% for fresh seeds.

**Table 2:** Seedling emergence percentages and index for fresh one-year-stored seeds of *Stipa bungeana* at sowing depths of 0, 1 and 5 cm.

Seed lots	Sowing depth (cm)	Emergence percentage	Emergence index
Fresh seed	0	18±2.9c	4.1±1.2d
	1	27±2.6ab	6.2±1.4c
	5	1±0.8e	0.2±0.2e
Stored seed	0	22±2.6bc	9.5±1.5b
	1	33±2.9a	14.2±1.2a
	5	8±1.5d	2.8±0.5d

Different letters within a column indicate significant differences (P<0.05, n = 10).

### Effect of burial on seed viability and dormancy

**Figure 6:**
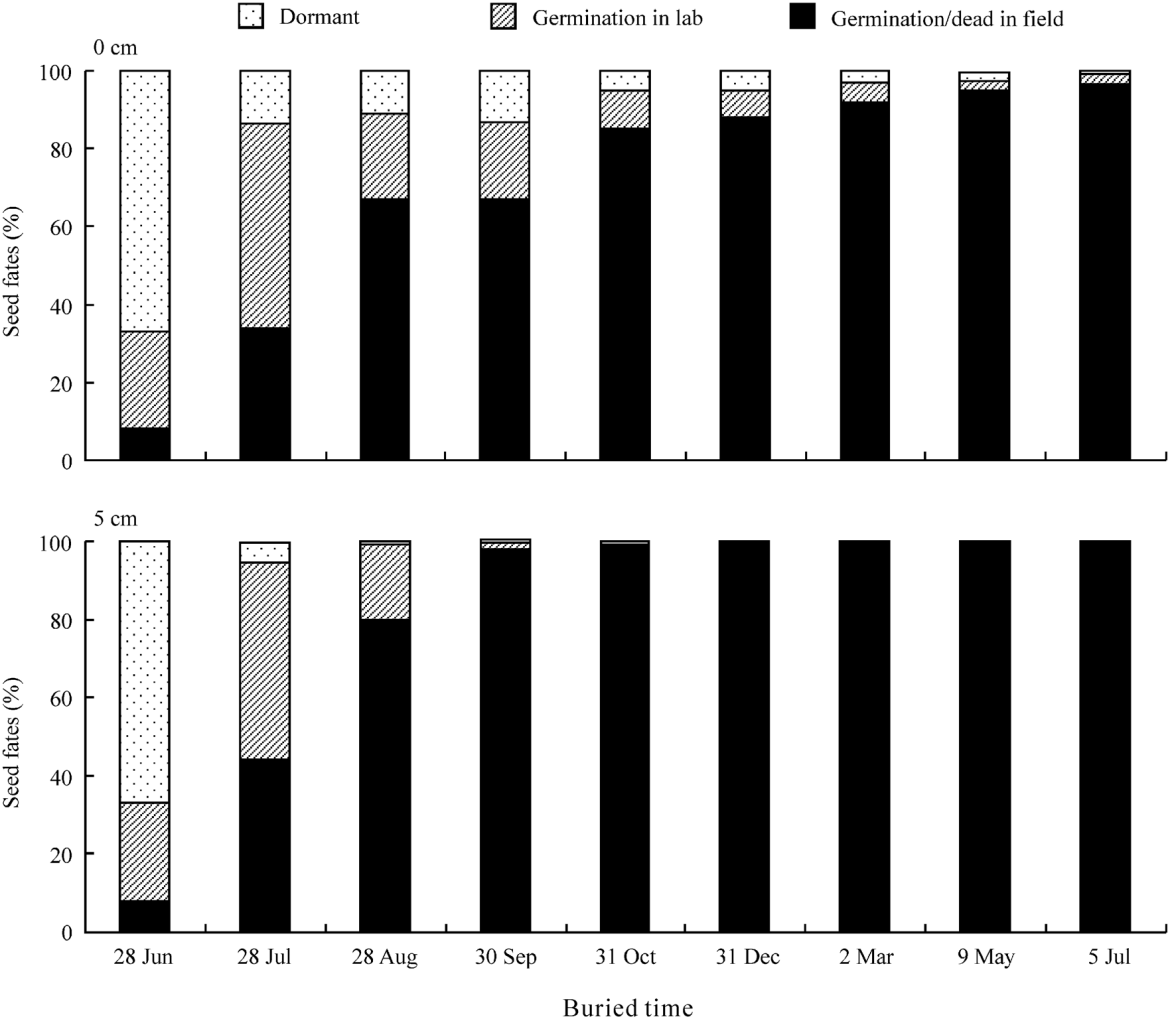
Fates of seeds in the field at 0 (surface) and 5 cm soil depths (n = 6).

Burial depth, burial duration and their interaction had a significant effect on seed viability (germinated seeds in the lab + dormant seeds) and on seed dormancy (Fig. 6, Table 3). After five months of burial at 5 cm, all seeds had lost viability, whereas 12% and 4% of those on the soil surface were viable after 5 and 12 months, respectively. Further, seeds buried at 5 cm lost dormancy more quickly than those sown on the soil surface. For example, almost 99% of seeds buried at 5 cm depth had lost dormancy after 3 months, while 87% seeds of those on the soil surface had done so.

**Table 3:** Two way ANOVA of the effects of burial time, burial depth and their interaction on seed viability and seed dormancy of *Stipa bungeana* (n = 6).

Source	Sum of Squares	df	F	P-value
Seed viability				
Burial time (BT)	24348	7	131.4	.000
Burial depth (BD)	1226	1	46.3	.000
BT * BD	638	7	3.4	.003
Seed dormancy				
Burial time (BT)	800	7	28.8	.000
Burial depth (BD)	224	1	56.3	.000
BT * BD	234	7	8.4	.001

### Temporal changes in soil seed bank size

**Figure 7:**
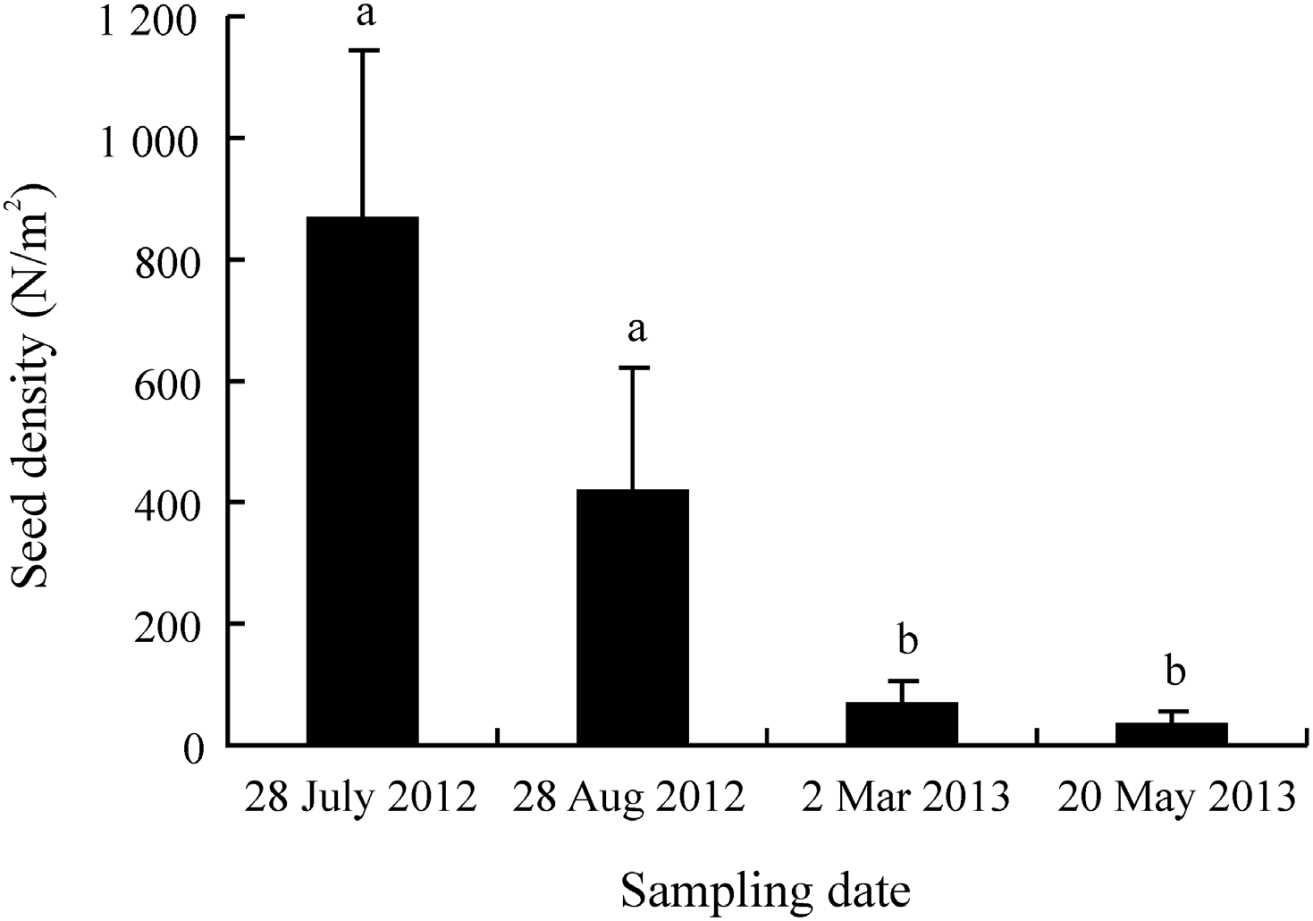
Seed density on different sampling dates in a *S. bungeana-*dominated grassland. Different letters indicate significant difference (P<0.05) among sampling dates (n = 16).

The size of the soil seed bank of *S. bungeana* declined with time and significantly so between August and March. The highest density of seeds in the seed bank was 869 m^−2^, in July, and the lowest density was 31 m^−2^, in May (Fig. 7).

## Discussion

### Seed dormancy and its underlying mechanism

Seed dormancy prevents or delays germination even under favorable conditions and spreads the risk of recruitment over time. It generally is accepted that seed dormancy plays a significant role in ensuring seed germination at the right time and in the proper sites to maximize the probability of successful seedling establishment [9], [14]. It is clear from the present study that *S. bungeana* seeds exhibit primary dormancy, since germination percentages were low at various combinations of light and temperature, and they were increased significantly by several dormancy breaking treatments. According to the seed dormancy classification system of Baskin & Baskin [8], [9], physiological dormancy can be caused by the tissues surrounding the embryo, by low growth potential of the embryo or by a combination of the two. The palea and lemma clearly play an important role in regulating germination of fresh *S. bungeana* seeds since their removal released dormancy completely. This is consistent with other studies that reported hulls imposed dormancy in other grass species, for example, *Stipa viridula* [15], *S. tenacissima* [16], *Leymus secalinus* [17], *L. chinensis* [18]–[20], *Hordeum spontaneum* [21], [22] and *H. vulgare* [23].

When hulls were removed from the dispersal unit of *S. bungeana* and then loosely reattached in their original position, seeds germinated to significantly lower percentage than those with hulls removed and not reattached. This suggests the possibility of the presence of germination inhibitors in the hulls, since the loosely-attached hulls should not have inhibited germination via mechanical restriction. Chemical germination inhibitors have been isolated from the hulls of grass seeds [24], [25]. ABA in hulls of *Leymus chinensis* inhibited germination of this species in vitro [26], [27]. Soluble germination inhibitors in the hulls of *Aegilops geniculata* [28], [29] may regulate germination response to amount of rain [30]. However, seed dormancy of *Stipa viridula* was not caused by chemical inhibitors in the palea and lemma since seeds with palea and lemma clipped on both ends without damage to the enclosed caryopsis and seeds with the lemma and palea removed germinated equally well [15]. The presence vs. absence of chemical inhibition of hulls on germination may vary among and within species [16], [24]. Huang *et al*. [31], Ma *et al.* [18], and He [32] suggested that the endosperm was responsible for seed dormancy in *Leymus racemosus* and *L. chinensis*, respectively. However, this obviously was not the case in *S. bungeana* since almost all fresh intact caryopses (with hulls removed) germinated.

Scarification with acids or alkalis has been shown to be effective in breaking seed dormancy in grasses [24], [25], and scarification with NaOH significantly increased germination of *S. bungeana* seeds. This may be due to damage to the hulls, thus decreasing their mechanical resistance to germination. Also, an increase in permeability of embryo covering tissues after treatment with NaOH may favor leaching of germination inhibitors from them. A combination of NaOH soaking and exogenous GA_3_ completely broke seed dormancy in *Leymus chinensis* [32].

ABA plays a role in the induction and maintenance of seed dormancy, whereas gibberellins (GAs) are associated with dormancy breaking and germination [33]–[35]. GA_3_ has been reported to stimulate germination of seeds of many grass species [36], and it does so by increasing the growth potential of the embryo [25], [33]. The small but significant increase in germination percentage of *S. bungeana* seeds by GA_3_ indicates that this plant growth regulator increased the growth potential of embryos in only some of the seeds to the point where the embryo overcame the mechanical restriction of its covering layers. The failure of fluridone, to promote germination suggests that ABA biosynthesis during imbibition may not be the primary cause of dormancy in *S. bungeana* seeds. Gianinetti & Vernieri [37] concluded that ABA is not the primary mediator of dormancy in imbibed rice seeds. However, a correlation between embryonic ABA level, sensitivity to ABA and hull imposed dormancy was found in barley [23].

Potassium nitrate is used extensively to break grass seed dormancy under laboratory conditions [38]. It alleviated the light inhibition of germination of *S. bungeana* seeds [27] and significantly increased germination of fresh seeds of this species (this study). Nitrate is one of the most ubiquitous inorganic ions in soils [39], and it can release seed dormancy and stimulate germination [9], [25], [36]. The much better regeneration of *Plantago lanceolata* in gaps than in closed vegetation may be attributed to higher nitrate level in gaps (0.2–1.1 mM) than that in closed vegetation (0.1 mM) [40]. Peaks in seedling emergence of *Capsella bursa-pastoris* in summer were correlated with increases in nitrate level in the soil [41]. Sensitivity of *Arabidopsis thaliana* seed to nitrate level at 20°C was highest in summer-early autumn when dormancy level in the seeds was low and expression of nitrate transporter 1.1 (*NRT1.1*) and nitrate reductase 1 (*NR1*) genes was highest [42].

Seed afterripening can be characterized by dormancy release and an increase of germination speed during dry storage [9], [43], [44]. Seeds of many grass species come out of dormancy during dry storage [16], [25], [36]. Dry storage for 4 months significantly increased germination percentage of *Stipa tenacissima* seeds [16], and the germination percentage of *S. bungeana* seeds increased significantly during dry storage for 1, 3 and 6 months at 5°C and 20°C. Effect of storage temperature on germination varies with the species [45]. A positive relationship between dormancy release rate and afterripening temperature has been found in seeds of some grass species [46]–[48]. Rate of afterripening of *Bromus tectorum* seeds was approximately the same at 20°C and 30°C [49]. Overall, however, storage temperature had only a small effect on germination of *S. bungeana* seeds, which germinated to 63% and 69% at 20°C in dark after dry storage for 6 months at 5°C and 20°C, respectively.

In sum, fresh seeds of *S. bungeana* have hull-imposed dormancy, and it can be released completely by removal of the hulls and in part by GA_3_, KNO_3_, NaOH scarification and dry storage. These results suggest that seeds of *S. bungeana* have non-deep physiological dormancy [8], as have been reported for seeds of many other species of grasses [9].

### Effect of seed dormancy on seedling emergence

On the Chinese Loess Plateau, caryopses of *S. bungeana* normally mature and are dispersed by the end of June (before rainy season). It is expected that seeds will germinate and seedlings become established in summer and autumn due to suitable temperature and precipitation for them to do so from July to September. Indeed, seedlings recruit mainly from July to September [7], [50]. Although they were dormant at the time of burial, and most seeds of *S. bungeana* that germinated from July to September (Fig. 6), and most of them were depleted from the soil during August and September (Fig. 7). There was no significant difference in the final percentage of seedling emergence in the field between stored and fresh seeds sown at 0 cm and those buried 1 cm (Table 2).

Although in final emergence percentage of stored and fresh seeds sown in the field did not differ, fresh seeds germinated much slower than stored seeds, indicating that primary dormancy delayed germination. The timing of germination has pronounced effects on subsequent survival and phenology of seedling, and, in turn, affect the reproductive output of adult plants [9], [14]. Water stress is one of the most important factors limiting seedling establishment in arid and semi-arid area. The long term (1957–2009) data showed that the humid index of study area ranked as September > August > July. Thus, seeds germinated in August or September will have an advantage for seedling growth and development with less water stress. However, severe winter conditions can over-ride this advantage through increasing seedling mortality. In present study, fresh seeds sown at the soil surface mostly emerged during 4–6 weeks after sown which corresponding the late July to mid-August after seed dispersal. However, stored seeds mostly emerged during 2–3 weeks after sown which corresponding the mid-July after seed dispersal. Thus, the delay in germination by seed dormancy of *S. bungeana* seems to be a compromise between water stress avoidance and seedling overwinter. This may provide an adaptive advantage of *S. bungeana* in arid environment.

Timing of seed germination largely depends on dormancy release which regulated by various environmental factors [9]. Dormancy of fresh seeds of *S. bungeana* buried in the soil was released more quickly than those on the soil surface (Fig. 6). There are at least three possible reasons for this difference. One is that nitrate in the soil may promote dormancy release (see above). Second, seeds were exposed to a higher level of hydration than those on the soil surface, which via microbial decay activity might have decreased mechanical resistance of the hull to embryo growth and thus germination. Third, seeds had high moisture content, which favored seed afterripening. Increasing the moisture content of rice seeds from 8% to 11% resulted in a 2.5-fold reduction in the storage period required for a given level of germination [51]. Seed dormancy release speed of *Lolium rigidum* increased as seed water content increased from 6% to 18% [48]. Increased dormancy release by burial may have ecological significance in that the seeds moisture conditions beneath the soil surface are more suitable for germination and subsequent seedling establishment than they are on the surface [7], [52].

### Effect of seed burial depth on seedling emergence and soil seed bank

The vertical distribution of a seed in the soil plays an important role in determining whether it remains dormant, germinates or dies [14]. Emergence percentage and speed (Table 2) were significantly higher for both fresh and stored seeds of *S. bungeana* buried 1 cm deep than they were for seeds sown on the soil surface (Table 2), probably due to the negative effect of light and water stress on germination [7]. Further, 16% seeds on the soil surface were dormant after one month of burial, but in only 5% of those buried at 5 cm (Fig. 6). Thus, buried seeds germinated to a higher percentage and speed than those on the soil surface.

All seeds buried 5 cm in soil and 88% of those on the soil surface on 28 June had either germinated or died by 31 December (Fig. 6). Further, the number of seeds in the soil seed bank decreased from 869 m^−2^ to 31 seeds m^−2^ between July and May (Fig. 7). Thus, our study indicates that only a small number of seeds produced by *S. bungeana* may have the potential to form a persistent seed bank, i.e. remain viable for ≥1 year in/on soil. Hou [50] reported that *S. bungeana* had a transient seed bank when seeds were hand-buried in nylon bags. However, *S. bungeana* formed a natural persistent seed bank in a typical prairie [53] and on eroded slopes [54], respectively, on the Loess Plateau.

### Practical implications

Fresh *S. bungeana* seeds exhibit primary dormancy, and thus seed pretreatment to release dormancy is important for quick establishment of *S. bungeana* plants. Although most seeds will germinate in 4–6 weeks after dispersal if soil moisture is suitable for them to do so, we recommended sowing one-year-stored (afterripened) seeds 1 cm deep to attain a uniform stand. An option is to pretreat fresh seeds with a 30% NaOH solution for 60 min before sowing them. Trampling by livestock during a short-term grazing period in early July immediately following seed dispersal may promote seed burial in the soil, which promotes germination. However, disturbance, such as by grazing, during the stand establishment period should be avoided in order to prevent seedlings from being injured or destroyed.
